# Temporal and regional variation in catch across an extensive coastal recreational fishery: Exploring the utility of survey methods to guide and assess spatio-temporal management initiatives

**DOI:** 10.1371/journal.pone.0254388

**Published:** 2021-07-21

**Authors:** Faith Ochwada-Doyle, Kate Stark, Julian Hughes, Jeffery Murphy, Michael Lowry, Laurie West

**Affiliations:** 1 New South Wales Department of Primary Industry & Environment, Wollongbar, New South Wales, Australia; 2 School of Biological, Earth and Environmental Science, University of New South Wales, Kensington, Australia; 3 University of Tasmania, Hobart, Tasmania, Australia; 4 New South Wales Department of Primary Industry & Environment, Mosman, New South Wales, Australia; 5 New South Wales Department of Primary Industry & Environment, Wollongong, New South Wales, Australia; 6 New South Wales Department of Primary Industry & Environment, Port Stephens, New South Wales, Australia; 7 Kewagama Research, Doonan, Queensland, Australia; University of Sydney, AUSTRALIA

## Abstract

As global research into recreational fishing gains momentum due to the pursuit’s biological, social and economic impacts, information on regional and temporal patterns of recreational exploitation will continue to enable objective assessment and development of management initiatives for exploited species. This paper demonstrates the utility of offsite survey methods in assessing spatial and temporal differences in recorded catches from a large, diffuse and heterogenous coastal recreational fishery. Using the estuarine recreational fishery that operates along the coast of New South Wales, Australia as a case study, survey data was employed to quantify annual (June 2013-May 2014) state-wide estuarine catch. Generalized linear mixed effects models were then applied to expanded catch estimates from surveyed households to examine the influence of zone and season on the kept and released numbers of snapper (*Pagrus auratus*), dusky flathead (*Platycephalus fuscus*) and bream (*Acanthopagrus* spp. complex comprised of *A*. *butcheri*, *A*. *australis* and their hybrids). For kept bream, significant differential seasonal effects were observed in all regions except the Mid-South Coast. For released bream, numbers were greatest in Sydney and during Summer and Winter. For kept snapper, the greatest harvest was recorded in the Mid-South Coast but season had no effect. Differential seasonal effects were found in each zone for released snapper. For kept dusky flathead, the greatest numbers were recorded in Sydney and the Mid-South Coast but season had no effect. We conclude by assessing some current spatial and temporal management initiatives in light of the uncovered patterns of recreational catch and consider the implications of these patterns in terms of future ecosystem-based management recommendations aimed at achieving ecological, social and economic sustainability in fisheries.

## Introduction

Research into recreational fisheries has rapidly gained momentum in recent decades [1–3]. In developed nations, in particular, an estimated 10.6% of the population fish recreationally [4, 5]. This significant participation rate has been one of the factors driving the exigency to monitor the social, economic and biological impacts and benefits of recreational fisheries [3, 6, 7].

In Australia, recreational fishing is thought to confer significant social benefits to over 3.4 million people (19.5% of population) who participate in the pursuit annually [7–9]. The economic contribution of the recreational fishing sector was valued at $AUD 2.56 billion in 2013 [10], which compares to an annual gross production value of $AUD 2.50 billion for the nation’s commercial fisheries and aquaculture during the same period [11]. In addition to these socio-economic aspects, harvests attributable to the recreational sector in many parts of Australia now rival or exceed commercial fisheries’ harvests for a number of species [9, 12–15]. This increases the potential for this sector to have significant biological effects on the abundance and size structure of key stocks [3, 16, 17].

As has been recognised in other developed nations [3, 6, 18], these characteristics continue to drive the comprehensive monitoring of recreational fishing in Australian waters to assist in developing resource management strategies that account for all sources of fishing mortality [7, 19]. A crucial component of this monitoring requires empirical investigation of spatial and temporal patterns in recreational exploitation which can, in turn, enable the development of spatially explicit and temporally relevant management strategies. Such assessments could also help in creating frameworks that are consistent with existing spatial and temporal management arrangements for commercial fisheries [see 20–22 for commercial fishery examples]. Considering the co-occurrence of many coastal recreational fisheries in Australia with commercial fishing sites, there exists a potential for competition among fisheries sectors [23, 24]. This conflict may be managed through spatial and temporal planning tools that aim to spatially segregate users or limit the impact of dominant sectors during biologically important seasons; which may also be better defined through spatio-temporal monitoring of recreational exploitation [23, 24].

This type of monitoring and assessment is particularly challenging for the diffuse and highly heterogenous coastal and marine recreational fisheries of Australia. These fisheries are often characterised by a concentration of effort near population centres and during certain times of the year; and often span large and variable environmental gradients [5, 15]. For such diverse and extensive fisheries, spatial and temporal management initiatives have often been considered and promoted as an effective means to reduce fishery impacts and conserve aquatic populations [25–31]. In some cases, however, such measures can be ineffective when applied without adequate consideration of the way recreational catches (both the harvested and released components) vary through space and time. For example, Chagaris et al. [32] highlighted the futility of implementing seasonal closures on a single species in multi-species recreational fisheries where a high discard mortality of the “protected” species could still occur during its closed season whilst anglers target different coexisting species. The effects of this type of implementation error might be minimized via an understanding of the temporal variation in recreational kept and released catch, which can elucidate variability in fisher behaviour. The inefficiencies of some spatial closures have similarly been discussed in terms of the unintended consequence of displacing recreational effort from a newly-protected region to neighbouring unprotected regions [33–35]. These latter studies highlight the need to anticipate the behavioural adaptations of anglers throughout the broader geographic extent surrounding the specific area being considered for closure [34], which could be facilitated through a better understanding of spatial variation in recreational catch. Effective assessment of existing spatial and temporal marine management initiatives and the planning of future initiatives therefore requires researchers and regulators to first understand the spatio-temporal nature of catch within their systems and then identify and retain persistently efficient fishing locations and seasons [20, 36].

The large, diffuse and heterogeneous nature of many recreational fisheries pose considerable challenges in the collection of reliable and representative catch and effort data over broad spatial and temporal scales [37]. Off-site methods like telephone-, diary-, mail- and internet-based surveys are therefore often employed to monitor and assess such fisheries in a cost-effective manner [8, 37–39]. In particular, telephone/diary-based surveys, with their characteristically larger sample sizes and/or higher response rates [38, 40], are recognized as best practice and used routinely for monitoring and assessing spatially and temporally expansive recreational fisheries in Australia [e.g.: 7, 8, 15, 41, 42] and internationally [e.g.: 39, 43–47].

Building on these ideas, this paper demonstrates the utility of telephone-diary survey methods in quantitatively assessing spatial and temporal differences in recorded catches from a large-scale and classically diffuse and heterogenous coastal recreational fishery. We do so by presenting an estuarine case study from the eastern Australian state of New South Wales (NSW) which has the nation’s greatest number of recreational fishers [8] and participation rates estimated at 11.9% [13]. The estuarine recreational fishery in NSW is the state’s largest in terms of catch and effort [13] and has a spatial extent of over 2,000 km of coastline [48]. This fishery is also diverse, occurring in bays and coastal rivers, with anglers using a multitude of methods (including line fishing, hand collection, potting, netting, spearing and/or diving) to capture over 100 different species from both shore- and boat-based platforms [13]. The state’s estuarine recreational fishery also overlaps with commercial fishing areas in some parts, with 9 out of 24 productive estuaries remaining completely open to commercial fishing [24]. Focusing on three finfish species that dominated the annual total recreational catch from estuarine waters and are also commercially important [49, 50] (snapper (*Pagrus auratus*), dusky flathead (*Platycephalus fuscus*) and bream (*Acanthopagrus* spp. complex comprised of *A*. *butcheri*, *A*. *australis* and their hybrids)), this study quantitatively examines the influence of zonal and seasonal differences on the harvested and released catch of each species. The regional and seasonal patterns of variation in catch identified through this type of study are a first step towards objectively assessing the effectiveness of current spatio-temporal management initiatives and developing ecologically and socio-economically effective future initiatives for key species across a diverse and extensive fishery [20].

## Methods

New South Wales Department of Primary Industries Approved this study involving human participants and provided written consent in the form of the DPI Fisheries Research Human Research Ethics Low Risk Application Form (INT20/76587). The data described within this manuscript focuses on three species within estuarine waters and represents a portion of the data collected for a study by West et al., 2015, which reported on recreational catch across all species in all waterbody types in NSW [13]. Unlike the West et al. (2015) report, this paper presents a novel and formal quantitative analysis of differences in recreational catch through space and time and considers the implications of these patterns for management.

### Study area

**Fig 1:**
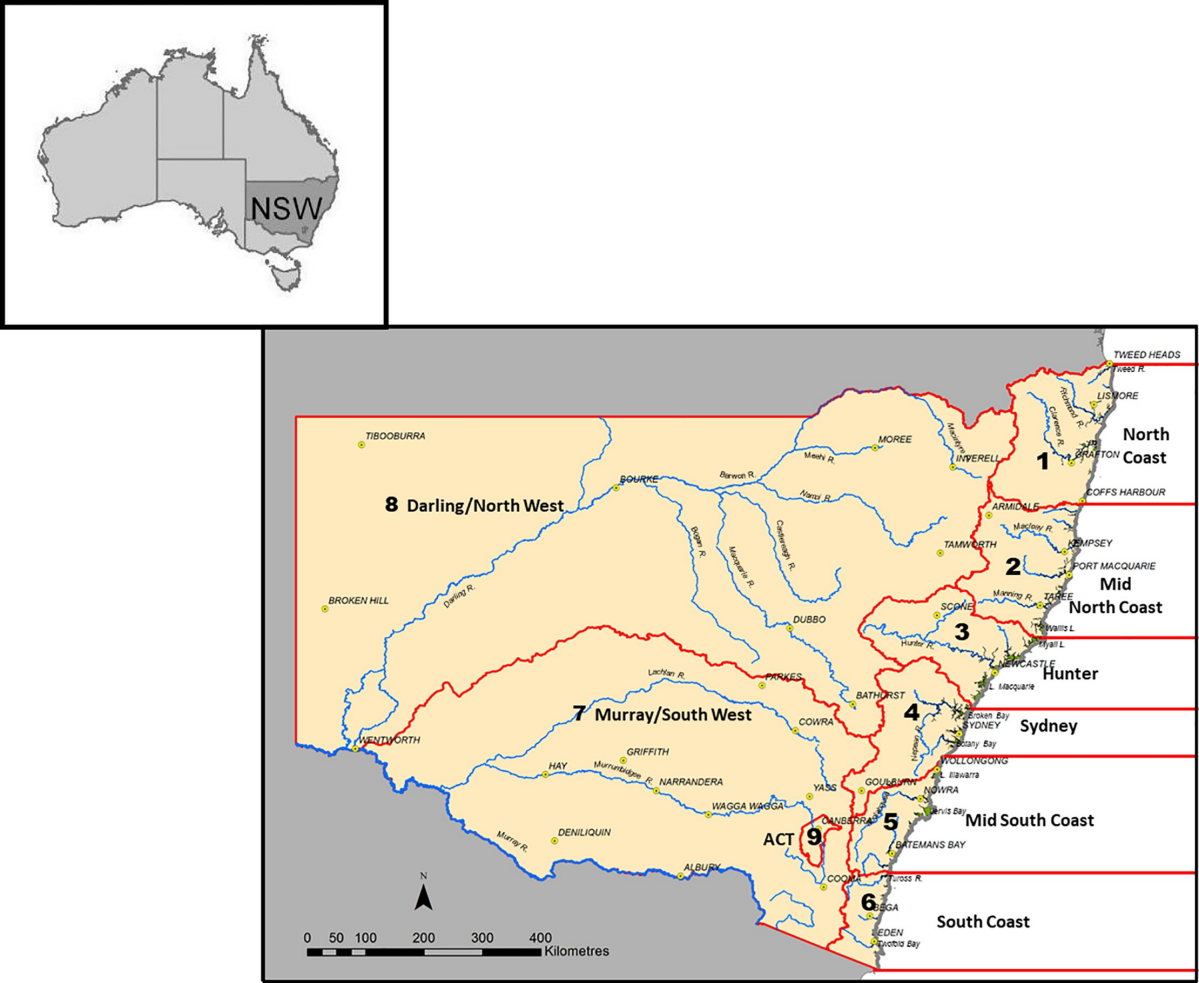
Relative size and location of the different fishing zones in New South Wales (NSW), Australia, including the North Coast, Mid-North Coast, Hunter, Sydney, Mid-South Coast and South Coast. Reprinted from (13) under a CC BY license, with permission from Jeff Murphy (NSW DPI), original copyright 2015.

This study considered estuary-based recreational fishing across the extensive NSW coastline (also known as the NSW Marine Estate [51]), which is comprised of around one million hectares of water and can be broadly divided into 6 geographic fishing zones: 1) North Coast; 2) Mid-North Coast; 3) Hunter; 4) Sydney; 5) Mid-South Coast; and 6) South Coast (Fig 1). These divisions were designed to accommodate major catchments, population centres and zones [8] (Fig 1).

### Data collection

Data on recreational catch was collected for fishing activity that occurred during the 2013/2014 period in NSW. The data was collected using an off-site telephone-diary survey methodology that was developed to provide cost-effective state-wide fishery information over a state-wide scale [8, 13, 52]. The telephone-diary approach involved a two-phase design, with the first component being an initial *Screening Phase* and the second an intensive *Diary Phase*. Each of these components are described in detail in West et al. 2015.

In brief, the *Screening Phase* was administered from March to May 2013 as a structured telephone interview of a randomly selected sample of NSW households (including households within the Australian Capital Territory (ACT)) [13] from the White Pages telephone directory. During the *Screening Phase*, 12,461 households were selected and contacted [13]. Of these households, 9,412 fully responded and 2,008 were identified as households that were eligible for the *Diary Phase* (See S4 Table) on the basis of at least one household member (aged 5 years or older) indicating an intention to fish in the ensuing 12 months [13]. Profiling information including age, gender and fishing avidity (measured as a function of estimated fishing frequency in the previous 12 months for residents aged 5 years or older) was also collected for each resident of a sampled household during the *Screening Phase* [13].

The *Diary Phase* comprised a longitudinal panel survey that monitored the fishing activity of all residents (aged 5 years or older) within recruited households between 1 June 2013 and 31 May 2014 [13]. A total of 1,681 households completed the *Diary Phase* [13]. With the aid of trained interviewer, a single diarist within each household that participated in the *Diary Phase* diarised basic information after each fishing event undertaken by any household resident (aged > 5 years). This information (see S5 Table) included date, location, start and finishing times and numbers of kept and released fish by species/species group [13]. Throughout the diary period, interviewers telephoned diarists repeatedly at regular intervals to conduct structured interviews that collected more detailed data on each fishing event, including fishing methods, fishing platform (boat-based or shore-based), water body type (freshwater, estuarine or oceanic), target species, and the numbers of kept and released fish by species/species group [13].

### Data analysis

Details on the analysis of the core survey data can be found in West et al., 2015. Briefly, this analysis was based on a stratified random survey design using single stage cluster sampling–with the household (n) representing the primary sampling unit and fisher residents within the household representing the secondary sampling unit. Expansion of information collected from each sampled household to population estimates relied on an integrated approach that calibrated against population benchmarks and adjusted for non-response [13, 53]. Expansion and calibration to attain population estimates of catch and associated standard errors (SEs) were completed using the *Survey* [54] and *recsurvey* [53] packages available in the statistical programming software *R* [55]. Refer to Lyle et al. (2010) and Lumley (2010) for equations.

Species-specific expanded estimates of total recreational catch from the survey were separated by waterbody (freshwater lakes/dams and rivers, saltwater estuaries, oceanic waters) to determine the most dominant finfish species (excludes baitfish) caught in each waterbody based on the total numbers. Snapper, dusky flathead and bream were the top-three finfish species/species groups caught in estuarine waters during the diary survey period.

Using the *glmer*.*nb()* command in the *lme4* package [56], generalized linear mixed-effects models (GLMMs) assuming a negative binomial distribution were utilised to examine the influence of the factors zone (levels: North Coast, Mid-North Coast, Hunter, Sydney, Mid-South Coast and South Coast; Fig 1) and season (levels: Winter, Spring, Summer and Autumn) on the expanded number of kept and released individuals of a given species [57, 58]. Since the primary sampling units in the study were randomly selected households and there was a possibility of some selected households catching a given species in more than one level of the factors zone and season, an inherent property of this survey data is that repeated catches across zones or seasons within a single household may be more similar to each other than catches in other households. Similarly, repeated catches across levels of each factor by a single individual (the secondary sampling unit) within a household may be more alike. The use of GLMMs enabled us to accommodate these properties (ie: lack of independence among primary and secondary sampling units) through inclusion of random-effects terms for individual households and persons [57, 59, 60]. Separate analyses were conducted for kept and released catches. All interaction terms and independent parameters were considered in the original model, which took on the general form of:

$$C=\beta_{0}+{\beta_{1}x}_{1,ij}+{\beta_{2}x}_{2,ij}{+\beta_{3}x}_{1,ij}x_{2,ij}+a_{j}+\varepsilon_{ij}$$ (1)

where *C* represents the kept or released catch; β_0_ the vertical intercept; β_1_ and β_2_ the regression coefficients for the independent parameters zone (*x*_1,*ij*_) and season (*x*_2,*ij*_) respectively; β_3_ the regression coefficient for the interaction term between zone and season; *a*_*j*_ represents a random variable; and, ε_ij_ represents the errors term [58, 61]. Models were initially fitted assuming both a Poisson or negative binomial distribution family. Akaike Information Criteria (AICs) and deviance values were then generated for these models using the *anova()* function and these were in turn used to select the model that had most appropriate distribution family, whereby the model with the lowest AIC and deviance values was deemed most appropriate [61]. These values consistently showed that the negative binomial distribution provided the best fitting models. The parameters in the original negative binomial model were then reduced iteratively towards parsimony [60, 62, 63]. Alternative models that consecutively excluded a single predictor parameter from the original model were compared based on AIC and deviance values (generated using the *anova()* function). If exclusion of a particular parameter lead to a higher AIC and deviance value, this meant that the model was not improved by that parameter’s exclusion and the parameter was therefore retained in the model [61, 64]. In doing so, the analyses retained the subset of predictor parameters that were most important in explaining variation in the kept or released catch [61] and therefore deemed omitted predictors as being of lesser influence. Note that partial tests have been shown to be an insufficient measure of the appropriateness of alternatives to a model [see 62, 65, 66], and were therefore only used to assess the influence of an independent parameter or interaction term once the final model had been chosen. For the final parsimonious models, Wald test (α = 0.05) was used to examine the null hypothesis that β_i_ = 0 [56, 61, 67] for a retained parameter or interactive term. Where the influence of zone and/or season were shown to be independently significant, Tukey’s post-Hoc tests (α = 0.05) were then used to compare the different levels of the significant parameter in a pair-wise manner with the interaction term from the original model being used as the error term [61]. Where the most parsimonious GLMMs detected a significant interaction between zone and season, a separate secondary series of GLMMs were applied to examine if catch was influenced by season at each level of zone separately using Wald test (α = 0.05) [56, 61, 68]. This examination was followed by Tukey’s post-Hoc tests (α = 0.05) whenever season was found to be significant at a particular level of zone.

## Results

**Fig 2:**
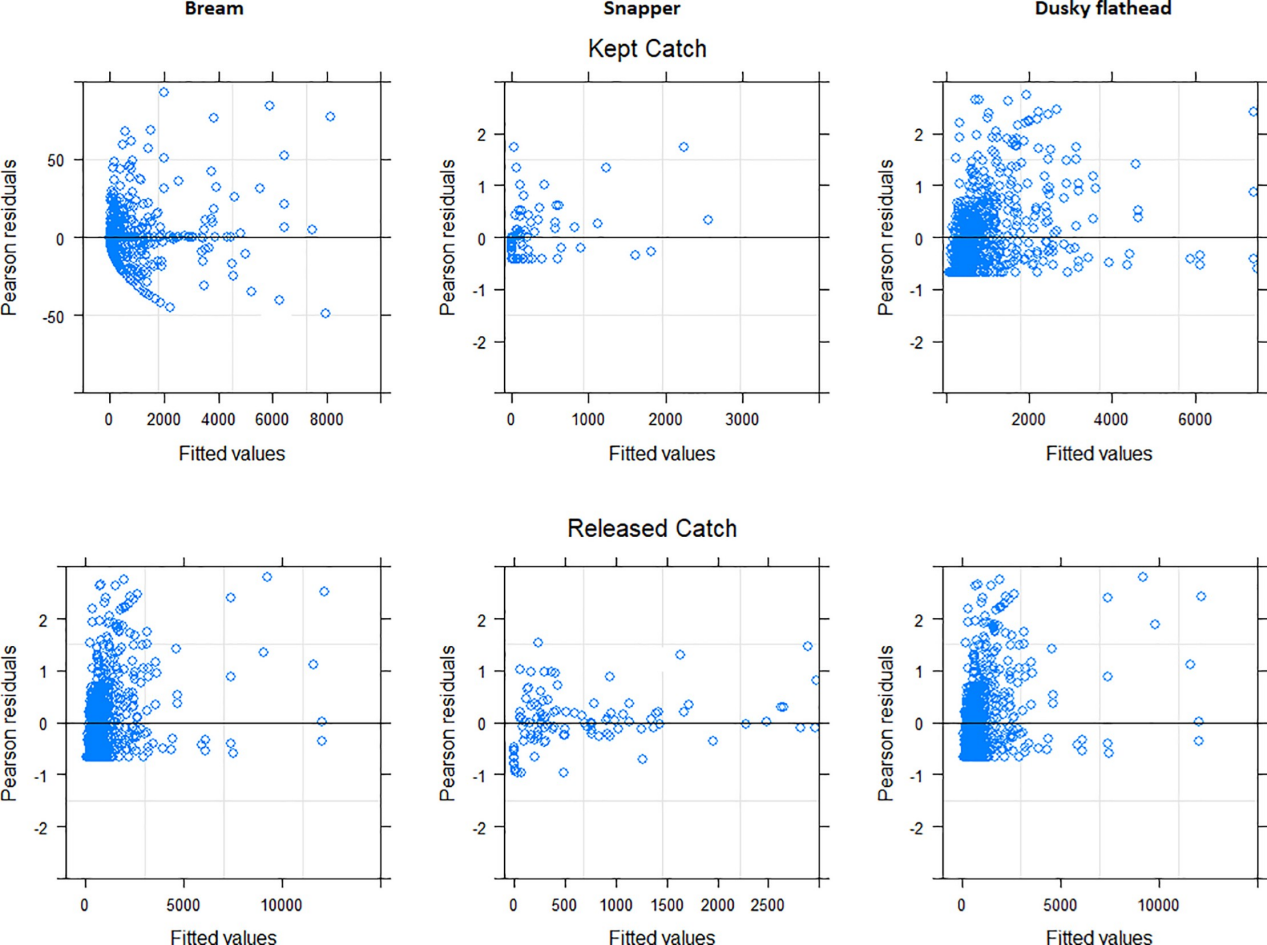
Pearson residual plots used to assess the fit of generalized linear mixed effects models that examined the influence of the categorical variables zone (6 levels: North Coast, Mid-North Coast, Hunter, Sydney, Mid-South Coast and South Coast) and/or season (4 levels: Autumn, Spring, Summer, Winter) on the number of estuary-caught bream, snapper and dusky flathead, that were kept and released from recreational fishing activity in NSW waters during 2013/14. Each model assumes a negative binomial distribution.

The estimated kept, released and total estuarine catch (across all species and for bream, snapper and dusky flathead) in NSW waters during the 2013/14 survey period are given in Table 1. For the final parsimonious GLMMz used to evaluate the influence of season and zone on the kept and released catch of bream, snapper and dusky flathead, scatter plots of Pearson residuals versus fitted values indicated that the overall spread of residuals around the zero line was fairly constant across all fitted values suggesting a satisfactory fit (Fig 2) [69]. In all cases however, the residuals were more densely concentrated around small fitted values and more sparsely concentrated around large fitted values.

**Table 1:** The estimated kept, released and total number of all species/species groups, bream, snapper and dusky flathead caught within estuarine waters in NSW during 2013/14.

	Catch	S. E	n
**Kept**			
All Species	4,489,951	767,728	443
Bream	497,270	102,393	213
Snapper	39,544	17,370	34
Dusky flathead	468,978	63,571	257
**Released**			
All Species	3,767,633	444,997	549
Bream	1,449,789	242,173	372
Snapper	422,370	131,910	78
Dusky flathead	549,344	81,176	259
**Total**			
All Species	8,257,585	1,056,511	617
Bream	1,947,059	291,294	417
Snapper	461,913	135,807	91
Dusky flathead	1,018,321	131,179	352

n is the number of households surveyed to get the estimate.

### Bream

The estimated catch of estuary-caught bream comprised 11.1% and 38.5% of the total (across all species) estuarine kept catch (in numbers) and released catch, respectively (Table 1). The total catch of bream in the North Coast, Mid-North Coast, Hunter, Sydney, Mid-South Coast and South Coast was 220,858 (± 50,330; n = 77) 345,584 (± 100,101; n = 91), 268,864 (± 54,866; n = 84), 556,089 (± 163,361; n = 70), 516,141 (± 143,420; n = 108) and 39,521 (± 12,549; n = 29). The seasonal total catch of bream was 366,404 (± 64,005; n = 119) for Winter, 320,119 (± 61,342; n = 148) for Spring, 680,676 (± 119,567; n = 214) for Summer and 579,859 (± 148,220; n = 167) for Autumn.

**Fig 3:**
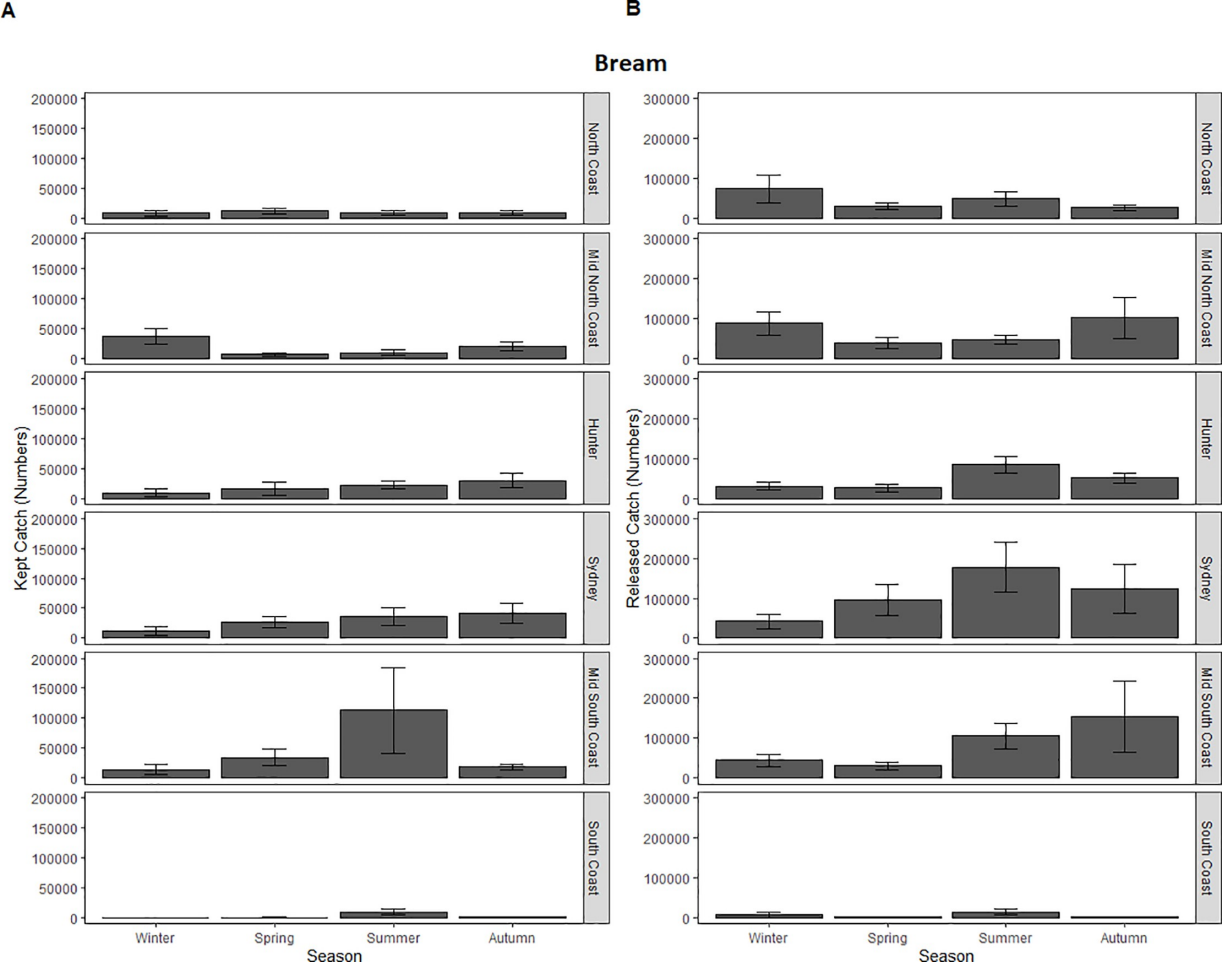
(A) Kept and (B) released catch of bream, snapper and dusky flathead in NSW estuarine waters during 2013/14 by zone and season. Error bars represent one standard error of the catch for each season.

AIC and deviance values for the full GLMM on kept catch and its reduced versions revealed that that the most parsimonious model was the full model that included the interactive term and both independent parameters. This retained GLMM detected a significant interaction between zone and season (Table 2A). A secondary series of GLMMs were therefore applied to examine how kept catch was influenced by season at each level of zone separately. For the North Coast, Mid-North Coast, Hunter, Sydney and South Coast the secondary GLMMs indicated that the regression coefficients for season was significant in terms of its influence on kept catch (P (>ChiSq) = <2.22E^-16*^). Within the North Coast, Tukey’s post-Hoc tests showed that the kept catch of bream in this zone was highest in Winter and lowest in Summer (Table 3A; Fig 3). Tukey’s post-Hoc tests showed that the kept catch of bream in the Mid-North Coast was highest in Winter but lowest in Spring (Table 3A; Fig 3). Tukey’s post-Hoc tests showed that the kept catch of bream in the Hunter was highest in Autumn and Spring but lowest in Summer (Table 3A; Fig 3). In the Sydney zone the kept catch was highest in Autumn but lowest in Winter (Table 3A; Fig 3). The Mid-South Coast was the only zone where season did not have a significant influence on kept catch (P (>ChiSq) = 0.22) (Table 3A; Fig 3). Within the South Coast, kept catch was highest during Spring and lowest during Autumn (Table 3A; Fig 3).

**Table 2:** Analysis of deviance tables showing the results of the generalized linear mixed effects models used to examine the influence of the categorical variables zone (6 levels: North Coast, Mid-North Coast, Hunter, Sydney, Mid-South Coast and South Coast) and/or season (4 levels: Autumn, Spring, Summer, Winter) on the number of estuary-caught (a) bream, (b) snapper and (c) dusky flathead that were kept and released from recreational fishing activity in NSW waters during 2013/14.

	Interaction term/Parameter	df	Chi Sq	Pr (>Chi Sq)
**(a) BREAM**				
Kept Catch	Zone	5	5464.30	< 2.20E^-16^*
	Season	3	14482.07	< 2.20E^-16^*
	Zone x Season	15	32271.28	< 2.20E^-16^*
Released Catch	Zone	5	24.57	1.26 E^-03^*
	Season	3	15.77	1.68 E^-04^*
**(b) SNAPPER**				
Kept Catch	Zone	5	27.54	4.47E^-05^*
	Season	3	1.82	0.61
Released Catch	Zone	5	31.33	2.63E^-06^*
	Season	3	15.38	1.52E^-03^*
	Zone x Season	11	26.29	5.88E^-03^*
**(c) DUSKY FLATHEAD**				
Kept Catch	Zone	5	15.50	8.41E^-03^*
Released Catch	Zone	5	6.81	0.23
	Season	3	2.76	0.43
	Zone x Season	15	11.01	0.75

The models, which assumed a negative binomial distribution, applied the Wald test (α = 0.05) to examine the null hypothesis that a β_i_ = 0, where β_i_s were the regression coefficients for each retained parameter or interaction term.

**Table 3:** The results of the Tukey’s post-Hoc test (α = 0.05) comparing different levels of season (4 levels: Autumn, Spring, Summer, Winter) in terms of the number of estuary-caught a) bream that were kept and b) snapper that were released within the North Coast, Mid-North Coast, Hunter, Sydney, Mid-South Coast and South Coast zones of NSW, Australia from recreational fishing activity during 2013/14.

	Comparison Pair	βi	S. E.	P-value
**(a) BREAM**				
Kept Catch for North Coast	Spring—Autumn	-0.25	0.02	< 2.00E^-16^*
	Summer—Autumn	-0.34	0.02	< 2.00E^-16^*
	Winter—Autumn	0.09	0.02	2.49E^-07^*
	Summer—Spring	-0.09	0.02	2.96E^-06^*
	Winter—Spring	0.34	0.02	< 2.00E^-16^*
	Winter—Summer	0.43	0.02	< 2.00E^-16^*
Kept Catch for Mid-North Coast	Spring—Autumn	-0.95	0.02	< 2.00E^-16^*
	Summer—Autumn	0.13	0.02	6.26E^-14^*
	Winter—Autumn	0.45	0.01	< 2.00E^-16^*
	Summer—Spring	1.07	0.02	< 2.00E^-16^*
	Winter—Spring	1.40	0.02	< 2.00E^-16^*
	Winter—Summer	0.32	0.01	< 2.00E^-16^*
Kept Catch for Hunter	Spring—Autumn	-0.01	0.02	1.00
	Summer—Autumn	-0.41	0.01	< 2.00E^-16^*
	Winter—Autumn	-0.33	0.02	< 2.00E^-16^*
	Summer—Spring	-0.40	0.01	< 2.00E^-16^*
	Winter—Spring	-0.32	0.02	< 2.00E^-16^*
	Winter—Summer	0.08	0.02	5.21E^-06^*
Kept Catch for Sydney	Spring—Autumn	-0.21	0.01	< 2.00E^-16^*
	Summer—Autumn	-0.03	0.01	3.98E^-04^*
	Winter—Autumn	-0.43	0.01	< 2.00E^-16^*
	Summer—Spring	0.18	0.01	< 2.00E^-16^*
	Winter—Spring	-0.22	0.01	< 2.00E^-16^*
	Winter—Summer	-0.40	0.01	< 2.00E^-16^*
Kept Catch for Mid-South Coast	Spring—Autumn	0.53	0.56	1.00
	Summer—Autumn	1.13	0.54	0.22
	Winter—Autumn	0.45	0.71	1.00
	Summer—Spring	0.60	0.55	1.00
	Winter—Spring	-0.08	0.71	1.00
	Winter—Summer	-0.68	0.72	1.00
Kept Catch for South Coast	Spring—Autumn	9.03	1.20	3.41E^-13^*
	Summer—Autumn	2.28	0.50	3.12E^-05^*
	Winter—Autumn	1.79	0.11	< 2.00E^-16^*
	Summer—Spring	-6.75	1.05	4.09E^-07^*
	Winter—Spring	-7.24	1.21	1.17E^-08^*
	Winter—Summer	-0.49	0.51	1.00
**(b) SNAPPER**				
Released Catch for Mid-North Coast	Spring—Autumn	-1.26	0.10	< 2.00E^-16^*
	Summer—Autumn	-0.41	0.09	1.33E^-05^*
	Winter—Autumn	-0.21	2.06	1.00
	Summer—Spring	0.85	0.05	< 2.00E^-16^*
	Winter—Spring	1.04	2.06	1.00
	Winter—Summer	0.20	2.06	1.00
Released Catch for Hunter	Spring—Autumn	-1.61	0.71	0.15
	Summer—Autumn	-0.52	0.49	1.00
	Winter—Autumn	1.22	0.69	0.46
	Summer—Spring	1.07	0.59	0.40
	Winter—Spring	2.83	0.91	0.01*
	Winter—Summer	1.75	0.70	0.07
Released Catch for Sydney	Spring—Autumn	0.19	0.28	1.00
	Summer—Autumn	1.48	0.24	3.05E^-09^*
	Winter—Autumn	0.88	0.23	7.95E^-04^*
	Summer—Spring	1.29	0.25	2.17E^-06^*
	Winter—Spring	0.69	0.30	0.15
	Winter—Summer	-0.60	0.29	0.21
Released Catch for Mid-South Coast	Spring—Autumn	-3.82	1.00	7.85E^-04^*
	Summer—Autumn	-2.47	1.07	0.13
	Winter—Autumn	-3.85	1.37	0.03*
	Summer—Spring	1.35	0.95	0.92
	Winter—Spring	-0.03	1.17	1.00
	Winter—Summer	-1.38	1.27	1.00
Released Catch for South Coast	Spring—Autumn	0.14	0.04	1.51E^-03^*
	Summer—Autumn	-0.41	0.02	< 2.00E^-16^*
	Summer—Spring	-0.55	0.04	< 2.00E^-16^*

AIC and deviance values revealed that the most parsimonious model for released bream was the one that retained season and zone as independent parameters but dropped the interaction term. The selected GLMM showed that both season and zone had a significant influence on the number of bream released (Table 2A; Fig 3). Tukey’s post-Hoc tests showed that significantly more bream were released in Sydney compared to each of the North Coast (β = 0.85 (± 0.24); P (>|z|) = 4.58E^-03*^), the Hunter (β = 0.67 (± 0.22); P (>|z|) = 0.04*), the Mid-South Coast (β = 0.74 (± 0.22); P (>|z|) = 0.01*) and the South Coast (β = 1.49 (± 0.33); P (>|z|) = 1.23E^-04*^) (Fig 3). Tukey’s post-Hoc tests also showed that significantly less bream were released in Spring compared to Summer (β = 0.42 (± 0.15; P (>|z|) = 0.04*) and Winter (β = 0.68 (± 0.18); P (>|z|) = 6.95E^-04^).

### Snapper

The estimated catch of snapper comprised 0.88% and 11.21% of the total (across all species) estuarine kept catch and released catch, respectively (Table 1). The total catch of snapper in the North Coast, Mid-North Coast, Hunter, Sydney, Mid-South Coast and South Coast was 618 (± 602; n = 1), 5,713 (± 3,339; n = 8), 112,570 (± 43,227; n = 24), 251,783 (± 105,699; n = 24), 72,945 (± 32,691; n = 31) and 18,283 (± 9,514; n = 10). The seasonal total catch of snapper was 36,129 (± 13,224; n = 19) for Winter, 43,426 (± 13,532; n = 27) for Spring, 261,192 (± 108,778; n = 47) for Summer and 121,166 (± 48,191; n = 32) for Autumn.

**Fig 4:**
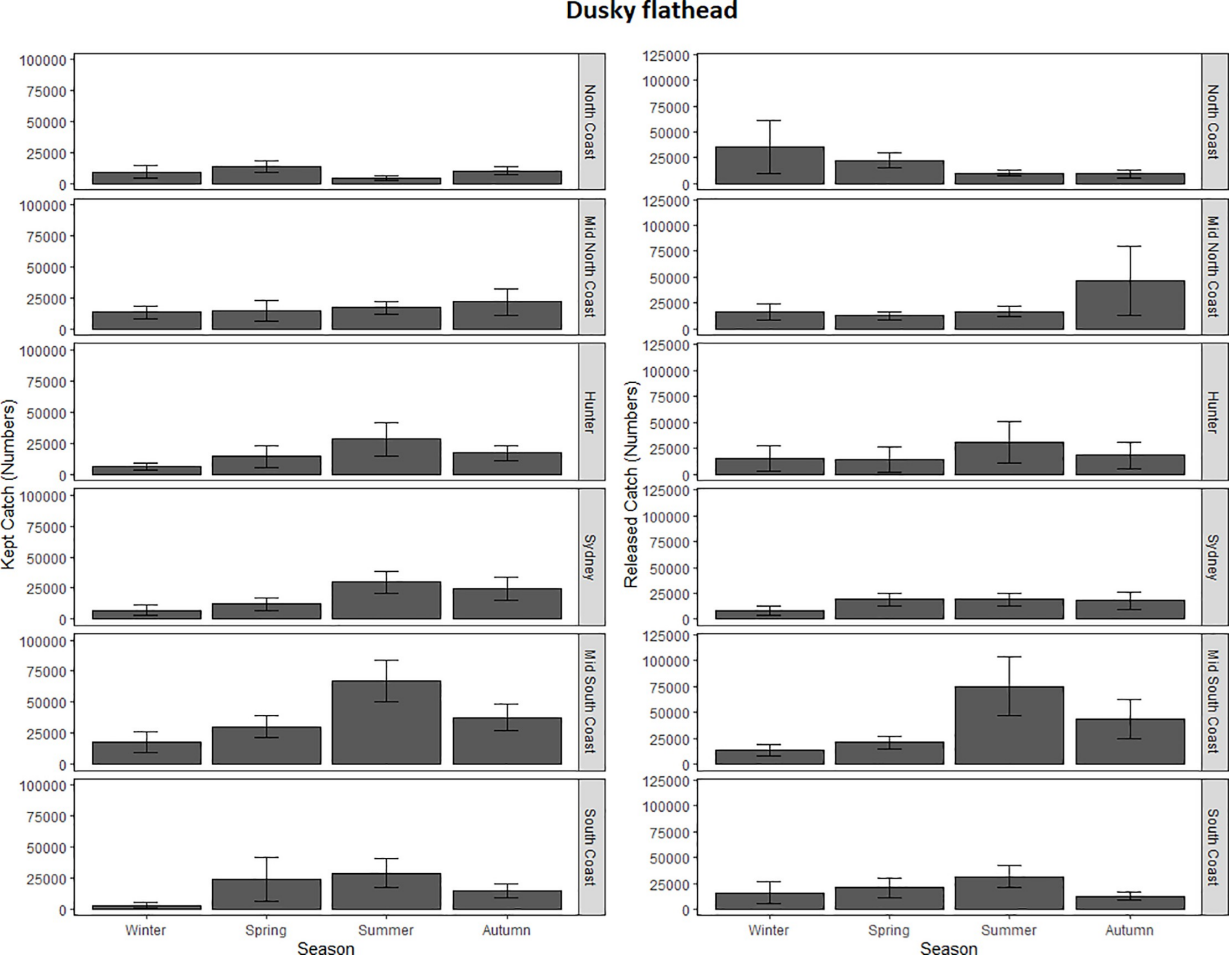


For the kept catch, the best fitting parsimonious model was the one that retained zone and season as parameters but omitted the interaction term. This model detected a significant effect for zone but not season (Table 2B; Fig 4). Based on the Tukey’s post-Hoc tests, significantly more snapper were kept from the Mid-South Coast compared to the Hunter (β = 8.20(± 1.92); P (>|z|) = 2.96E^-04^). There were also fewer snapper in the South Coast compared to the Mid-South Coast (β = -8.10 (± 2.21); P (>|z|) = 3.71E^-03^). Significantly less snapper were kept from Sydney compared to the Mid-South Coast (β = -8.39 (± 1.95); P (>|z|) = 2.55E^-04^).

The North Coast was not included in the analyses of released snapper data as there were no records of this species being released within this zone. The most parsimonious model for released snapper was the one that retained both independent parameters as well as the interaction term and this model detected a significant interaction (Table 2B; Fig 4). A secondary series of GLMMs were therefore applied to examine how released catch was influenced by season at each level of zone separately. In each zone, the effect of season was significant (P (>ChiSq) = <2.22E^-16*^ - 1.51E^-03*^). Tukey’s post-Hoc tests showed that the released catch of snapper in the Mid-North Coast was highest in Autumn but lowest in Spring (Table 3B; Fig 4). Tukey’s post-Hoc tests showed that the released catch in the Hunter was highest in Winter but lowest in Spring (Table 3B; Fig 4). In the Sydney zone, the released catch was highest in Summer and lowest in Autumn (Table 3B; Fig 4). Within the Mid-South Coast the estimated catch was highest during Autumn and lowest during Winter (Table 3B; Fig 4). For the South Coast, kept catch was highest during Spring and lowest during Summer (Table 3B; Fig 4).

### Dusky flathead

The estimated catch of dusky flathead comprised 10.45% and 14.58% of the total (across all species) estuarine kept catch and released catch, respectively, in numbers (Table 1). The total catch of dusky flathead in the North Coast, Mid-North Coast, Hunter, Sydney, Mid-South Coast and South Coast was 117,185 (± 35,091; n = 63), 159,256 (± 50,963; n = 66), 143,395 (± 71,834; n = 52), 138,935 (± 31,929; n = 47), 306,722 (± 66,563; n = 107) and 152,828 (± 46,679; n = 50). The seasonal total catch of dusky flathead was 163,176 (± 37,107; n = 80) for Winter, 359,545 (± 57,938; n = 184) for Summer, 276,265 (± 57,224; n = 131) for Autumn and 219,335 (± 40,042; n = 120) for Spring.

**Fig 5:**
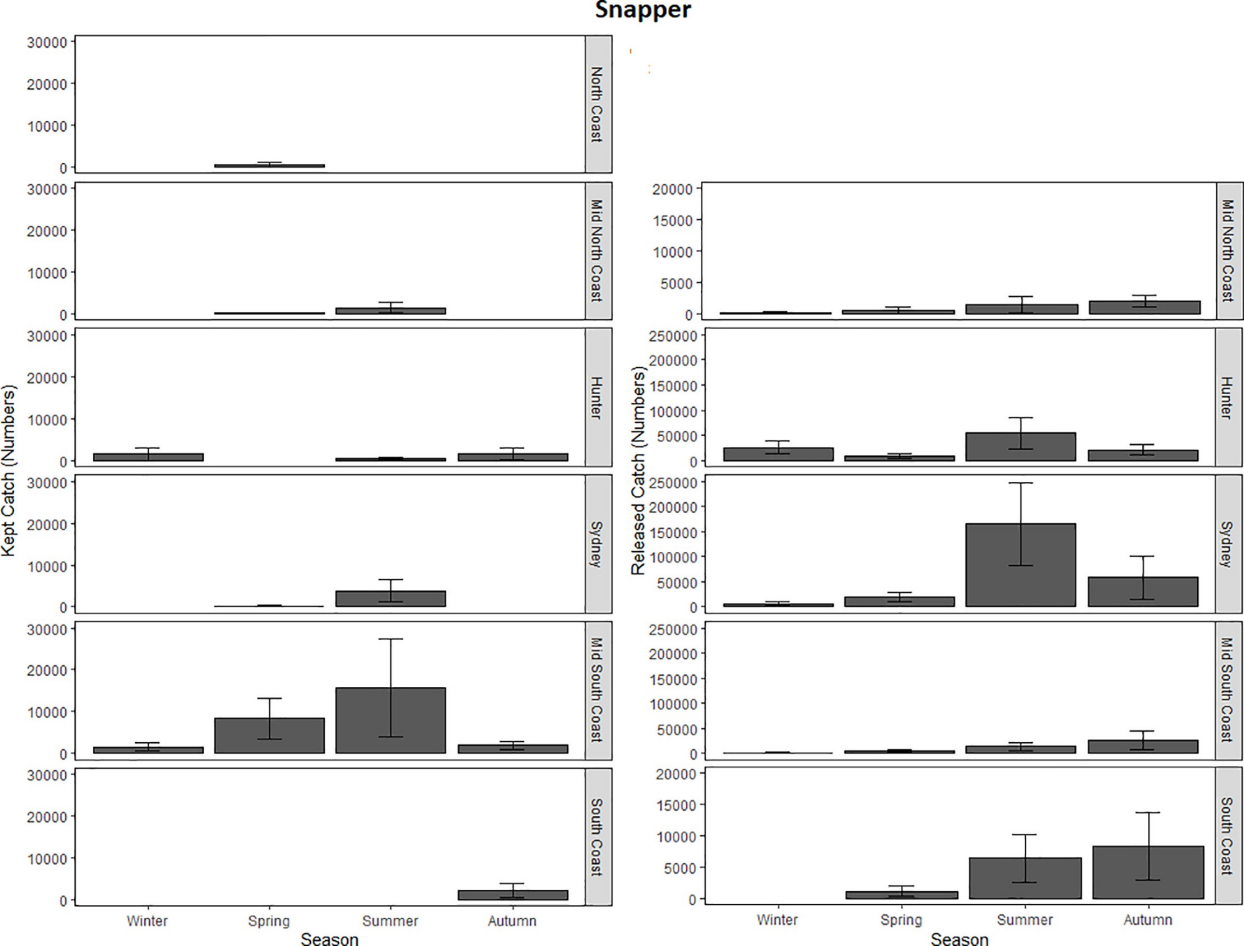


For dusky flathead’s kept catch, the most adequate model only retained zone. This model detected significant spatial effects (Table 2C; Fig 5) and Tukey’s post-Hoc tests showed that significantly fewer dusky flathead were kept in the North Coast compared to Sydney (β = 1.18 (± 0.34); P (>|z|) = 7.90E^-03*^) and the Mid-South Coast (β = -0.95 (± 0.28); P (>|z|) = 1.18E^-02*^).

Based on AIC and deviance values, the most parsimonious model for the released dusky flathead catch was the full model that retained both independent parameters and the interaction term, but this model did not detect any significant effects (Table 2C; Fig 5).

## Discussion

The notion that spatial and temporal restrictions to fishing are an effective means to manage and conserve fisheries resources remains a prevailing paradigm in coastal and marine research and management [20, 25–31]. In the context of managing recreational fisheries to maintain angling quality and prevent population collapse, spatial and temporal management restrictions are often implemented alongside, or as an alternative to, gear, size and quota limitations [70]. However, many authors have questioned the effectiveness of spatial and temporal management arrangements and have suggested that quality in the design and implementation of such initiatives is often lost in a push towards quantity [32, 71–73]. The outcomes of such spatial and temporal arrangements could be vastly improved with more specific spatio-temporal information on patterns of recreational catch. By elucidating this information for three key species harvested in the recreational fishery that operates in estuaries along the coast of NSW, the current study will play an important role in optimizing the placement and timing of future spatial and temporal management initiatives to maximize their ecological effectiveness as well as their socio-economic benefits to the recreational fishery.

### Comparison of current findings and previously documented patterns

Although the overall kept catch of bream in estuarine waters appeared slightly higher in the Mid-South Coast, a statistically significant interaction between zone and season was detected with differential seasonal effects observed in North Coast and the Mid-North Coast where kept catch was highest in Winter; the Hunter and Sydney where kept catch was highest in Autumn, and the South Coast where kept catch was highest in Spring. For released bream, zone and season had a significant effect with the highest numbers being released in Sydney and during Summer and Winter. For snapper, only zone had a significant influence on harvested numbers with the greatest numbers being recorded in the Mid-South Coast. Differential seasonal effects were found in each zone for released snapper with the greatest release numbers recorded in Autumn for the Mid-North Coast and the Mid-South Coast, Winter for the Hunter, Summer for Sydney and Spring for the South Coast. For kept dusky flathead, only zonal effects were significant with the greatest numbers recorded in Sydney and the Mid-South Coast. Significant spatial and seasonal effects were not detected for released dusky flathead. For all species/species group, there were clear differences in the seasonal patterns observed for kept catch and released catch. Species-specific seasonal differences in the size frequency distribution or natural abundance may have meant that there were more small fish or larger abundances in general during specific seasons. This may have resulted in anglers releasing more fish during these seasons to adhere to size and bag limit regulations, compared to seasons when fish were larger and perhaps less abundant.

For each species/species group, Table 4 summarises previous findings on their spatial and temporal patterns of distribution and/or abundance as described in the reviewed literature of both fishery-dependent and fishery-independent studies. The current results generally reiterate those described for estuarine waters in the previously published works. Although there are few published studies on the broad regional patterns of distribution and/or abundance of bream in NSW, there is some anecdotal evidence that this taxon migrates northward in Winter to spawn [74], which might explain the pattern of higher kept catches within the North Coast and Mid-North Coast during Winter. The spatial distribution of past offshore recreational catches and commercial landings for snapper also differ slightly to the observations reported here, whereby the highest catches were formerly recorded in the state’s north [75, 76]. Although previously reported commercial catches of snapper also suggest a slight interactive influence of season and region, the patterns are slightly different from those uncovered here with commercial landings being highest between Winter and Spring in northern latitudes and then slightly higher in Summer compared to Winter in southern latitudes [75].

**Table 4:** Findings on the spatial and temporal patterns of distribution and/or abundance as described in reviewed literature for the bream species complex, snapper, and dusky flathead.

Species/taxon	Spatial Distribution/Abundance	Temporal Distribution/Abundance
**BREAM**	*A*. *butcheri*: is an obligate estuarine species, found in brackish and fresh waters, with a range that extends from southern NSW (around 36.028° S, 150.32° E) down to Victoria, South Australia and into Western Australia as far north as Shark Bay (25.783° S, 113.299° E) [84]. *A*. *australis*: is a marine species that occurs in estuarine and oceanic waters and inhabits a wide range of habitats including rocky reefs, sandy areas, seagrass, mangroves and structures such as marinas and jetties [78]. Its distribution extends from central Queensland (23.171° S, 150.957° E) to southern NSW (around 36.012° S, 150.167° E) [84]. Surprisingly, there are few published fishery-dependent or fishery-independent studies examining the broad regional patterns of distribution and/or abundance of *Acanthopagrus* spp in NSW.	Across NSW, the highest commercial catch of bream have been recorded during Autumn and Winter [97]. A study conducted in two estuaries in North Coast (Clarence River and Richmond River) found that recreational catch rate of *A*. *australis* was highest in the Autumn and Winter months [98]. Although few *A*. *butcheri* were recorded in their onsite survey of recreational fishing, Steffe and Chapman [99] found that the catch of *A*. *acanthopagrus* was higher during Autumn and Summer within an estuary located in Hunter (Lake Macquarie). In a similar study within an estuary in South Coast (Tuross Lake), Steffe et al. [100], found that catch rates for this species were highest during Winter and Spring in 1999/2000 and highest in Autumn and Summer in 2003/2004. In both studies, however, the difference among seasons was noted as not statistically different.
**SNAPPER**	*P*. *auratus* is common in coastal and offshore waters from the Capricorn Group in Queensland (23.450° S, 151.956° E) around the southern coast of Australia to Coral Bay in Western Australia (23.144° S, 113.771° E) [101]. Adult *P*. *auratus* occur across a variety of habitats, particularly rocky reefs, whilst juveniles mainly inhabit estuaries and embayments [78, 102]. In a study that used baited remote underwater video systems (BRUVS) to measure the abundance of species in NSW Marine Parks, Malcolm et al. [103] found that the abundance (measured as Max N) of *P*. *auratus* was most similar and highest in Marine Parks located in the North Coast and Hunter compared to a Marine Park located in the Mid-South Coast. Catch rates for *P*. *auratus* in the coastal recreational charter fishery of NSW were shown to be highest in northern coastal waters between Wooli (29.89° S) and Coffs Harbour (30.31° S) [104]. A recent analysis of the species’ movement based on a tag and recapture dataset from Australia’s east coast showed that although movements over large distances were rare, individuals that did move were more likely to do so in a northerly direction [79]. Furthermore, about 60% of the commercial catch of this species in NSW comes from the northern third of the state [75]. This pattern of higher catches in the north are mirrored by the offshore recreational fishing sector [75, 76].	A model-based analysis of *P*. *auratus* BRUV data from New Zealand found that overall relative densities were ~2–3 times greater in Autumn than in Spring for juvenile sized snapper [105]. Catch rates for *P*. *auratus* in the coastal recreational charter fishery of NSW were shown to be highest in Autumn and Winter [104]. Autumn and Summer were shown to be the seasons with the highest catches of this species in a survey-based general assessment of an estuarine recreational fishery in the Hunter [99]. In the far north of NSW, historical commercial catch data shows that the largest landings of this species occur between Winter and Spring with few snapper landed during Summer [75]. In southern latitudes, the difference between Winter and Summer commercial landings diminishes and the pattern becomes somewhat reversed [75].
**DUSKY FLATHEAD**	*P*. *fuscus* is found in estuaries and nearshore coastal water from Cairns, Queensland (16.919° S, 145.778° E) to the Gippslands Lakes in Victoria (38.000° S, 147.650° E) [106]. This species occurs over sand, mud, gravel and seagrass and can inhabit estuarine waters up to the tidal limit (https://www.dpi.nsw.gov.au/content/fisheries/recreational/saltwater/sw-species/dusky-flathead). A scientific observer program used to quantify the composition of catches and discards in the gillnet fishery for *P*. *fuscus* in three NSW estuaries found that the retained catch of legal sized fish was not significantly different between an estuary located in Mid-North Coast (Wallis Lake) and two estuaries located in the Sydney zone and the Mid-South Coast (Tuggerah Lake and Lake Illawarra) [83, 106].	In a study conducted in two estuaries in the North Coast (Clarence River and Richmond River), West & Gordon [98] found that catch rate of *P*. *fuscus* was only slightly higher in mid- to late Spring compared to other periods. Based on their onsite survey of recreational fishing, Steffe and Chapman [99] found that the catch of *P*. *fuscus* was higher during Autumn and Summer compared to Winter and Spring within an estuary located in the Hunter (Lake Macquarie). In a similar study within an estuary found in South Coast (Tuross Lake), Steffe et al. [100], found that catch rates for this species were highest during Summer & Spring. For both onsite surveys however, the observed seasonal differences were not statistically significant.

When compared to the NSW commercial catch data for the 2013/14 period (NSW DPI unpublished data), the current study on recreational catch also reveals some differential spatial and temporal patterns among the sectors. The retained estuarine commercial catches (in terms of weight) for the bream complex were highest in the Northern Coast (consists of the North Coast and Mid-North Coast) whilst our overall recorded recreational harvest numbers were highest in the Mid-South Coast. For snapper, kept catches from both sectors were highest in the Southern Coast (which consists of the Mid-South Coast and South Coast). Commercial catches of dusky flathead were highest in the Northern Coast whilst our recorded recreational harvest was lowest in the North Coast. Seasonal differences also existed among the two sectors. The commercial catch of bream and snapper were highest in Winter and Autumn, respectively; compared to a higher overall recreational harvest during Summer for bream and seasonal homogeneity in recreational harvest for snapper. The commercial catch of dusky flathead was highest in Winter compared to seasonal homogeneity in recreational harvest.

### Spatio-temporal management initiatives; some examples of recommendations and assessments

Our results for snapper show that estuaries in the Mid-South Coast represent areas of persistently high recreational harvest. Within NSW, this zone has the highest density of estuarine “Recreational Fishing Havens” (RFHs), which are designed to improve the quality of recreational fishing and enhance angling opportunities [30]. The regional patterns revealed here suggest that these spatial management arrangements may be having some success in terms of maximising recreational snapper catches. However, considering the higher number of major coastal rivers and estuaries in the Southern Coast of NSW (46% of the total number in NSW [77]), the observed patterns for snapper may simply be a relic of higher estuarine abundances and/or fishing opportunities in the south due to greater habitat availability for juveniles [78]. Whilst “no-take” areas within NSW Marine Parks are not specifically designed as a spatial management tool for populations of a single species or species group, they nonetheless represent small areas that can affect the populations in and around Marine Parks within the North Coast (the Cape Byron & Solitary Islands Marine Parks), Mid-North Coast (Lord Howe Island Marine Park), Hunter (Port Stephens/Great Lakes Marine Park), Mid-South Coast (Jervis Bay and Batemans Marine Parks) since they are closed to recreational fishing [49, 51]. Accordingly, these areas have been shown to be ecologically effective in increasing the abundance and size of snapper [79–82]. To offset the social and economic impact of “no-take areas” on the angler experiences within Marine Parks, artificial reefs could be deployed outside of “no-take areas” as a means of maintaining recreational fishing opportunities for snapper, particularly within the high harvest Mid-South Coast zone.

Spatial closures are used alongside gear-based temporal restrictions for the commercial sector to manage the bream fishery in NSW [50]. Some of these spatial closures fall under the “no-take areas” of NSW Marine Parks, but also include specific temporal closures that only apply to the estuarine commercial gillnet fishery, whereby, fishing is prohibited during most of summer in two estuaries (Wallis Lake and Tuggerah Lake) and from spring to late autumn in three estuaries (Smiths Lake, Lake Illawarra and St Georges Basin) [83]. If spatially differentiated seasonal closures were being considered for the bream estuarine fishery to create recreational management frameworks that were more consistent with existing commercial arrangements, the application of specific spatio-temporal closures could be required. For example, seasonal closures during Spring, Summer or Autumn in the North Coast and Mid-North Coast, during Summer, Spring or Winter in the Hunter and Sydney zones and during Summer, Autumn or Winter in the South Coast could potentially enable recreational fishing to continue during seasons in which anglers have a higher chance of experiencing high catches. In applying such measures, however, careful consideration of the species’ biology would be necessary due to the fact that bream populations within estuaries have been shown to be reproductively active during the extended period between late-Winter to mid-Summer [84].

For dusky flathead, spatial closures and gear-based temporal restrictions similar to those described for bream are used to manage the species’ fishery [85]. Our results for dusky flathead harvested demonstrate that estuarine recreational catches are relatively static across seasons. A marine stock enhancement program for dusky flathead is currently under development in NSW and aims to increase recreational fishing opportunities [86]. Based on our findings, estuaries within the lower harvest North Coast, Mid-North Coast and Hunter zones may benefit most from these future stock enhancement initiatives in terms of boosting socio-economic returns for the recreational fishery

To provide a better understanding of the interannual consistency or variation in the spatio-temporal patterns revealed here and therefore enable any of the above management initiatives to evolve where necessary, off-site telephone/diary surveys of the NSW recreational fishery must be repeated on a regular interannual basis to provide a time series of recreational catch estimates. The differences among seasons that were reported here, for example, are not a true index of overall seasonal patterns of catch since sampling only took place over one year as opposed to multiple years. It is important to note that broad-scale offsite survey methodologies such as those used here can be limited in terms of accuracy and precision when it comes to estimating the catch of species that are caught infrequently or as part of a “niche” recreational fishery [8, 38]. In such cases, the future development of customised complimentary surveys (including on-site surveys) that target rare or niche species should be investigated and future analytical research to improve the accuracy and precision of survey estimates is highly recommended. Another short coming of the present survey design is its propensity to predominantly sample less avid anglers (those who fish infrequently) and inadequately sample avid anglers due to the use of a general population sampling frame (i.e. White Pages directory) [87]. This particular shortcoming is reflected in our data since the residuals plots for all our models show densely concentrated residuals around small fitted values (i.e.: estimates of low annual catches) and sparsely distributed residuals around large fitted values (i.e.: estimates of high annual catches). This pattern is probably due to a majority of individuals within surveyed households being anglers with low avidity and therefore catching fewer fish. The more avid high catch anglers, on the other hand, are under-represented in our data. Recreational fishing or boating licence databases can offer more targeted sampling frames that adequately sample avid anglers and therefore improve precision [15, 38, 87]. Such databases are currently being trialled in NSW as a next step in the evolution of recreational fishery assessments [87]. Since this study was only based on fishery-dependent data, inferences on how recreational catches influence the standing stocks of the species studied are inherently limited. It may therefore be of benefit to compliment future recreational assessments with simultaneous fishery-independent assessments [88, 89]. It is acknowledged, however, that the cost of undertaking state-wide fishery-independent assessments may be prohibitive.

## Conclusion

This paper clearly highlights the utility of offsite telephone/diary survey designs in profiling spatial and temporal patterns of exploitation across a large, diffuse and heterogeneous recreational fishery. When such designs comply with the requirements of random and representative sampling, as was the case here, they have been shown to be a cost-effective option for monitoring and assessing fishery impacts to some extent [39]. More importantly, this paper demonstrates how an understanding of spatial and seasonal variation in the catch of key species can inform the assessment of current and future management initiatives. Such assessment can enable development of management options that are not only beneficial to the ecological sustainability of individual target species, but also include components to enhance socio-economic outcomes for the recreational fishery.

Although there are many other significant pressures that must be mitigated to lessen impacts on coastal fish stock and therefore achieve population conservation, fishing constitutes a significant extractive enterprise that must be regulated for sustainability [90]. The recent growth of the recreational fishing sector in developed countries has occurred alongside a global increase in the prominence of holistic forms of natural resource management [3, 4, 6, 91–94]. As these shifts continue, the type of work described here will become increasingly important due to its ability to facilitate ecosystem-based fisheries management by accounting for the triple bottom line criteria of achieving ecological, social and economic sustainability in recreational fisheries [94–96].

## Supporting information

S1 Table This is the raw and weighted data analysed in the manuscript for bream. In each species’ table, PersonID is a unique identifier given to each angler in a surveyed household; HouseholdID is a unique identifier given to each household sampled for the survey; AppendixName refers to the common name of the species caught; NKept is the raw number of fish of the species in question kept by a person during the 2013/14 period; NReleased is the raw number of fish of the species in question released by a person during the 2013/14 period; Season is the season during which the person kept/released the stated number of fish; RegionName is the fishing zone in which the person kept/released the stated number of fish; NTotal is NKept + NReleased; CalibratedP2_Weight is the final weight calculated for a person following the application of a sampling weight and response propensity and calibration adjustments; NKept_Weighted is NKept * CalibratedP2_Weight which gives the expanded estimate of kept catch for the whole population represented by a person; NReleased_Weighted is NReleased * CalibratedP2_Weight which gives the expanded estimate of released catch for the whole population represented by a person; and, NTotal_Weighted is NTotal * CalibratedP2_Weight which gives the expanded estimate of total catch for the whole population represented by a person.
(CSV)

S2 Table This is the raw and weighted data analysed in the manuscript for snapper. In each species’ table, PersonID is a unique identifier given to each angler in a surveyed household; HouseholdID is a unique identifier given to each household sampled for the survey; AppendixName refers to the common name of the species caught; NKept is the raw number of fish of the species in question kept by a person during the 2013/14 period; NReleased is the raw number of fish of the species in question released by a person during the 2013/14 period; Season is the season during which the person kept/released the stated number of fish; RegionName is the fishing zone in which the person kept/released the stated number of fish; NTotal is NKept + NReleased; CalibratedP2_Weight is the final weight calculated for a person following the application of a sampling weight and response propensity and calibration adjustments; NKept_Weighted is NKept * CalibratedP2_Weight which gives the expanded estimate of kept catch for the whole population represented by a person; NReleased_Weighted is NReleased * CalibratedP2_Weight which gives the expanded estimate of released catch for the whole population represented by a person; and, NTotal_Weighted is NTotal * CalibratedP2_Weight which gives the expanded estimate of total catch for the whole population represented by a person.
(CSV)

S3 Table This is the raw and weighted data analysed in the manuscript for dusky flathead. In each species’ table, PersonID is a unique identifier given to each angler in a surveyed household; HouseholdID is a unique identifier given to each household sampled for the survey; AppendixName refers to the common name of the species caught; NKept is the raw number of fish of the species in question kept by a person during the 2013/14 period; NReleased is the raw number of fish of the species in question released by a person during the 2013/14 period; Season is the season during which the person kept/released the stated number of fish; RegionName is the fishing zone in which the person kept/released the stated number of fish; NTotal is NKept + NReleased; CalibratedP2_Weight is the final weight calculated for a person following the application of a sampling weight and response propensity and calibration adjustments; NKept_Weighted is NKept * CalibratedP2_Weight which gives the expanded estimate of kept catch for the whole population represented by a person; NReleased_Weighted is NReleased * CalibratedP2_Weight which gives the expanded estimate of released catch for the whole population represented by a person; and, NTotal_Weighted is NTotal * CalibratedP2_Weight which gives the expanded estimate of total catch for the whole population represented by a person.
(CSV)

S4 Table These are the questions asked of each household during the Screening Phase of the survey. (PDF)

S5 Table These are the questions asked of each household during the Diary Phase of the survey. (PDF)
