# Supplementary material for: Temporal and regional variation in catch across an extensive coastal recreational fishery: Exploring the utility of survey methods to guide and assess spatio-temporal management initiatives
Source: PLoS One. 2021 Jul 21;16(7):e0254388. doi: 10.1371/journal.pone.0254388 (PMC8294510; doi:10.1371/journal.pone.0254388)
Supplement: S4 Table — (PDF) [file pone.0254388.s004.pdf]

<u>NOTES: QUESTIONNAIRE DESIGN - NSW RECREATIONAL FISHING SURVEY (2013-14)</u>

Attached are the questionnaires for two key survey components:-

1) Screening Survey (Sects. A & BC): this questionnaire structure establishes whether people/households have recreationally fished in the previous 12 months and/or intend to fish in the coming 12 months; and

2) Diary Survey (Questions and Fishing Events [answers]): regular data collection over a 12 month period for each time a household member goes recreational fishing/etc., whether they caught anything or not.

Since the mid 1990's, many routine design 'conventions' have been employed by consultant staff to simplify the interviewers' work – as per the following examples from Sect. A of the Screening questionnaire:-

a) any text in lower-case bold format is to be precisely read-out by the interviewer – see Q2(a);

b) whereas, upper-case text in 'normal' format refers to interviewer instructions – see latter part of Q3 ;

c) however, all *italicised* text relates to "sequence guides" – see answer categories 1 and 2 of Q3 ["*(go to Sect. B)"*].

Although the Diary Survey generally employed these conventions, an additional design feature is that all respondents were routinely provided with a 'survey kit'. This included a specially-designed Diary Card (see attachment), plus a species identification booklet with many 'expert' images from Roger Swainston (anima.net.au).

Importantly, we believe that the <u>extremely high response rates</u> and data quality achieved in this project (and so many others using this approach) are the direct result of customising the survey design with a <u>specific focus</u> on the respondents (whether they fish a lot, a little ... or not at all).

.

SURVEY OF RECREATIONAL FISHING IN NSW/ACT, 2013/14 – SCREENING SURVEY©

A: Administrative Section, Introduction and Initial Screening

IN CONFIDENCE

(Mail-merge ..) **N99999** **WAVE #** **I/VER** **SUBURB/LOCALITY** **(02) 8999 9999** **2nd No/Mobile**

Day/Mth	Time	Result (C/NC/NA)	Day/Mth	Time	Result (C/NC/NA)	Appointments/other	Response Report	
							Fully responding	1
...... /......	....	....	...... /......	....	....	....	Full refusal	2
...... /......	....	....	...... /......	....	....	....	Part refusal	3
...... /......	....	....	...... /......	....	....	....	Full non-contact	4
...... /......	....	....	...... /......	....	....	....	Part non-contact	5
...... /......	....	....	...... /......	....	....	....	Language/communic.	6
...... /......	....	....	...... /......	....	....	....	Number disconnected	7
...... /......	....	....	...... /......	....	....	....	Business number	8
...... /......	....	....	...... /......	....	....	....	Fax/email line (perm.)	9
...... /......	....	....	...... /......	....	....	....	Holiday home/no UR's	10
...... /......	....	....	...... /......	....	....	....	Other (specify)	11
...... /......	....	....	...... /......	....	....	....	....	
...... /......	....	....	...... /......	....	....	....	....	

Q1. INTRODUCTION: **Good morning/etc. .... from (the) NSW Fisheries (Department) .... research about recreational fishing ....** (IDENTIFY 'ARA'; FEW QUICK QUESTIONS/LESS THAN 1 MIN. FOR MOST PEOPLE; CLIENT = FISHERIES NSW – DEPARTMENT OF PRIMARY INDUSTRIES; KEWAGAMA RESEARCH = CONSULTANT; NUMBERS RANDOMLY SELECTED FROM 'WHITE PAGES' TELEPHONE BOOKS; VOLUNTARY/ CONFIDENTIAL SURVEY; ABOUT HOW MANY PEOPLE GO FISHING/OWN BOATS, "THAT KIND OF THING")

Q2(a) **(Firstly), thinking back over the last 12 months …. has any member of your household done any recreational fishing in Australia … whether they caught anything or not?**

Yes	*(go to Sect. B)*	1
No		2

(b) **(And during this time has any household member done/… <u>or</u>) any <u>other</u> kind of recreational fishing like crabbing, prawning, spearfishing … or even collecting oysters or aquarium fish?**

Yes	*(go to Sect. B)*	1
No		2

Q3. **(And) thinking about the coming 12 months, how likely is it that a member of your household will do <u>any kind</u> of recreational fishing, crabbing etc.? Would you say …** (READ OUT UNTIL TERMINATED; *UNSURE NOT VALID AS 'INITIAL' PROXY RESPONSE IN Q'S 2-4, ARRANGE PERSONAL INTERVIEW/CALL-BACK ETC)

**Very likely?**	*(go to Sect. B)*	1
**Quite likely?**	*(go to Sect. B)*	2
**Not very likely?**		3
**Not at all likely?**		4
UNSURE*		5

Q4. **(And) does anyone in your household own a boat of any kind … including canoes, jet skis … or commercially-used boats?** (INCL. PARTIAL/ CORPORATE OWNERSHIP; 'GUNWALE' RULE)

Yes	1
No	2

Q5(a) **(And) could you tell me how many people live in your household?** (HOW MANY PEOPLE THESE ANSWERS APPLY TO; ALL AGES/USUAL RESIDENTS) Total Usual Residents ☐

(b) **(And of these, how many are male/s)?** (BY OBSERVATION, IF ONE PERSON; RECORD IN TOTAL AND DEDUCE FEMALES)

(c) PROBE FOR NO. OF MALES/FEMALES BY AGE <u>OR</u> AGE GROUP E.G. **And what are the ages of the males?**

	<5yrs	5-14	15-29	30-44	45-59	60plus	Total
Males	☐	☐	☐	☐	☐	☐	☐
Females	☐	☐	☐	☐	☐	☐	☐

Q6. THANK AND TERMINATE INTERVIEW

COMMENTS: ....

....

....

© 2013 Fisheries NSW (DPI) and Kewagama Research

B: Household Structure and Fishing Activity

SAMPLE NO: N

Q1. INTRO AS <u>APPROP</u>. (FEW DETAILS ABOUT H'HOLD NOW; THEN Q'NS ABOUT EACH PERSON'S FISHING … <u>OR</u> [E.G.] ASK Q3 ONWARDS FOR 'ARA' THEN AGE/THEN ASK FOR OTHER H'HOLD MEMBERS) **… Could you tell me all the people who usually live there, starting with the head of the household/oldest person?** (PROBE/ RECORD; IDEALLY ANY <5 YRS LAST; NAMES NOT ESSENTIAL; CIRCLE PERSON NO/S SPOKEN WITH)

**PERSON NO:**		**1**	**2**	**3**	**4**	**5**	**6**	**7**	**8**	**9**
(a) NAME/IDENTIFIER (H, W, S, D, ETC)		....	....	....	....	....	....	....	....	....
(b) **What was … age last birthday?** (OR AGE GROUP: <5, 5+, 15+, 30+, 45+, 60+)		☐	☐	☐	☐	☐	☐	☐	☐	☐
(c) SEX (observation)	Male	1	1	1	1	1	1	1	1	1
	Female	2	2	2	2	2	2	2	2	2

SG2.
- *FOR EACH RESPONDENT AGED 5 YEARS OR MORE, GO TO Q3*
- *OTHERWISE (I.E. 'OUT OF SCOPE'), NO MORE QUESTIONS*

Q3(a) **Thinking back over the last 12 months, have you done any recreational fishing at all in NSW (or the ACT) … whether you caught anything or not?**	Yes *(go to Q4)*	1	1	1	1	1	1	1	1	1
	No	2	2	2	2	2	2	2	2	2
(b) **(And during this time, have you done/… <u>or</u>) any <u>other</u> kind of recreational fishing in NSW (or the ACT) like crabbing, prawning, spearfishing … or even collecting oysters or aquarium fish?**	Yes	1	1	1	1	1	1	1	1	1
	No	2	2	2	2	2	2	2	2	2
Q4. **(And) during the last 12 months, have you done any kind of recreational fishing in <u>another state or territory</u> … or crabbing, spearfishing, etc. (again, whether you caught anything or not)**	Yes	1	1	1	1	1	1	1	1	1
	No	2	2	2	2	2	2	2	2	2

SG5.
- *IF FISHED/ETC <u>ANYWHERE IN AUST</u> IN PREV. 12 MONTHS (CODE 1 IN Q'S 3 [a/b] OR Q4), GO TO Q6*
- *OTHERWISE, GO TO Q7*

Q6. **(And) in the last 12 months, on how many separate days did you do <u>any kind</u> of recreational fishing, crabbing etc. (in Australia)? Would you say …** (READ OUT UNTIL TERMINATED)	**Less than 5 days?**	1	1	1	1	1	1	1	1	1
	**5 to 9 (days)?**	2	2	2	2	2	2	2	2	2
	**10 to 14 (days)?**	3	3	3	3	3	3	3	3	3
	**15 to 19 (days)?**	4	4	4	4	4	4	4	4	4
	**20 or more (days)?**	5	5	5	5	5	5	5	5	5
Q7. **(And) thinking about the coming 12 months, how likely is it that you will do <u>any kind</u> of recreational fishing, crabbing etc? Would you say …** (READ OUT UNTIL TERMINATED; *PROXY UNSURES/CALL-BACK ETC)	**Very likely?**	1	1	1	1	1	1	1	1	1
	**Quite likely?**	2	2	2	2	2	2	2	2	2
	**Not very likely?**	3	3	3	3	3	3	3	3	3
	**Not at all likely?**	4	4	4	4	4	4	4	4	4
	UNSURE*	5	5	5	5	5	5	5	5	5

SG8.
- *IF ANY INTENDING FISHER (CODE 1 OR 2 IN Q7), GO TO Q1, SECTION C*
- *OTHERWISE, GO TO <u>Q4</u>, SECTION C*

C: Diary Survey Invitation (Intending Fisher Households)

Q1. EXPLAIN TO MAIN/EACH INTENDING FISHER, AS APPROP: **As you can see, the survey we've just done will provide information about the number of people who fish/etc … but to get a more complete picture on recreational fishing, we'd like your help with the second stage of our research … in which we're asking people about their fishing (crabbing etc) over the next 12 months (starting 1st June). (Don't worry) it's not hard and it doesn't take much time to do. What happens is … we send you out a survey kit and I/we'll call you every now and then, to get information about any fishing/etc. that you might do. Would you be willing to take part in this research?** (STRONGLY ENCOURAGE – AT LEAST LOOK OVER KIT BEFORE DECLINING; EASY/INTERESTING; IMPORTANT SCIENTIFIC INFO. – WHETHER YOU FISH A LOT OR A LITTLE; COMPLETELY CONFIDENTIAL)

PERSON NO/S (INTENDING FISHERS ONLY)	☐	☐	☐	☐	☐	☐
Yes	1	1	1	1	1	1
No (*Go to Q5, if all intending fishers decline*)	2	2	2	2	2	2
Unsure (BUT KIT TO BE SENT)	3	3	3	3	3	3

Q2(a) PROBE NAME/S FOR COVER SHEET
(OR PER SECT. B, OR LATER AT EXPL. I'VIEW) ……….. ……….. ……….. ……….. ……….. ………..

(b) MAIL DETAILS FOR KIT (CIRCLE TITLE OR INSERT E.G. Dr.)

Mr Ms Mrs Miss ……………………… ……………………………………………
(Initials/First Name) (Surname/Family Name)

Address: ……………………………………………………………………………………………

Suburb: ……………………………… ………………. NSW/ACT Postcode: ………..

Q3. THEN EXPLAIN: **you should receive your survey kit by the last week in May/etc …… soon after that I/we'll call you to quickly run through it all with you.** (EXPLAIN DIFFERENT INTERVIEWER, IF APPROP). **Are we likely to catch you then/etc ….** (PROBE: BTC/AWAY INFO; AVOID FIRM APPOINTMENTS; IF ONLY ONE PHONE NO., PROBE FOR MOBILE/OTHER NO., THEN ASK/RANK ALL NO/S)

BTC/AWAY/OTHER INFO: ………………………………………………………………………………………

………

………

………

………

………

………

………

RANK PHONE NUMBERS (1, 2, X, ETC): Main No: ☐ 2nd No: ☐ Other: ☐ ………………………………………

Q4. (JUST ONE FINAL QUESTION …) **Does anyone in your household own a boat of any kind… including canoes, jet skis … or commercially-used boats?** (INCL. PARTIAL/CORPORATE OWNERSHIP; 'GUNWALE' RULE)		
	Yes	1
	No	2

Q5. (REMIND DIARIST H'HOLDS ABOUT NEXT CALL), THEN THANK AND TERMINATE INTERVIEW

IMPORTANT: PLEASE COMPLETE RESPONSE REPORT IN SECT. A, THEN STAPLE TO THIS Q'AIRE. FOR EACH DIARY SURVEY HOUSEHOLD, TRANSCRIBE ALL 'HEADER' INFORMATION TO DIARY SURVEY COVER SHEET – INCLUDING SNUM, NAMES, AGES/ETC. AND BTC/AWAY DETAILS.
