# Supplementary material for: Temporal and regional variation in catch across an extensive coastal recreational fishery: Exploring the utility of survey methods to guide and assess spatio-temporal management initiatives
Source: PLoS One. 2021 Jul 21;16(7):e0254388. doi: 10.1371/journal.pone.0254388 (PMC8294510; doi:10.1371/journal.pone.0254388)
Supplement: S5 Table — (PDF) [file pone.0254388.s005.pdf]

SURVEY OF RECREATIONAL FISHING – 2013/14

DIARY SURVEY INTERVIEW – QUESTIONS FOR EVENT SHEETS©

N.B. PROXY INTERVIEWS GENERALLY TO BE CONFINED TO NIL/JOINT ACTIVITY, CHILDREN ETC.

A **IDENTIFICATION OF DAYS/DATES**

- LAST TIME WE SPOKE WAS ... (ALERT: LAST EVENT OR ANY 'OPEN' EVENT FROM PREV. CONTACT)
- **Since then, have you done any kind of recreational fishing/crabbing etc ... including any days when you didn't catch anything?** (PROBE/STORE NO. OF SEPARATE DAYS) **(And has any other family/ household member done any fishing/etc since then?)** (INCL. NON-DIARISTS, AGE 5+ AT SCREENING)
- IF ONE FISHING DAY ONLY, GO TO PART B BELOW
- (IF MORE THAN ONE FISHING DAY) **So where did you go fishing/etc on these days? Did you fish/etc anywhere else during this time?** (PROBE/STORE REGION INFO/SPLITS ETC). **(And) did you fill out your diary card for each of these days? Have you got it there?** (NOW GO TO PART B BELOW)
- IF NO EVENT, GO TO PART C

B **FOR EACH DATE/EVENT** (CHRONOLOGICAL ORDER; KEY PROBE: **What did you do first/next?**)

Q'n No:

2 **So (that/the first/the next) day was ... ?** (RECORD START DATE OF EACH EVENT; IF END DATE IS DIFFERENT, INSERT NUMBER OF ADDITIONAL DAYS [PASSIVE GEAR USUALLY] - LEAVE BLANK IF SAME; ***SPLIT EVENT*** FOR EACH SEPARATE DAY UNLESS PASSIVE GEAR USED OR OTHER CONTINUOUS FISHING THROUGH MIDNIGHT)

3 **(And did you fill out your diary card for this day? Have you got it there?) (NO *SPLIT EVENT*)**

4, 5 **What did you do that day? (Where did you go fishing/etc that day? Did you fish/etc anywhere else?)** (PROBE/RECORD FISHING SITE AND IF ANY DOUBT, EXTRA LOCALITY INFO, E.G. NEAREST TOWN; CLASSIFY REGION CODE LATER FROM LOOK-UP FILE; ***SPLIT EVENT*** IF DIFFERENT FISHING SITES (SUBSTANTIVE) OR REGION CODES; 'MAINLY' RULE FOR TRUE BORDELINE CASES, I.E. WHERE SEPARATE CATCH AND EFFORT DATA IMPRACTICAL)

5 (IF SUB-REGION NOT EVIDENT) **So where in …** (REGION) **were you fishing?** (PROBE/APPLY …)

CODE 1 VS 2: OCEAN WATERS > OR < 5KM (RESPONDENT PERCEPTION) SEAWARD/AWAY FROM THE MAINLAND/COAST;

CODE 2 VS 3: KEY DEFINITION = 'OUTSIDE' VS 'INSIDE' THE MOUTH OF THE RIVER/HARBOUR/ETC; REFER 'LOOK-UP' FILE FOR KEY ESTUARY BOUNDARIES (E.G. SYDNEY HARBOUR); FOR OTHER COASTAL AREAS PROBE: OPEN SEA VS. 'ENCLOSED' BAY/ETC (RESP. PERCEPTION)

CODE 3 VS 4: FOR COASTAL RIVERS/ESTUARIES ASK, IF NEEDED: **Were you fishing/etc in saltwater or fresh?** (RESPONDENT PERCEPTION; PLUS ALERT FOR SW VS. FW SPECIES TARGETS)

THEN ASK: **Did you fish anywhere else that day in …** (REGION)**?** (***SPLIT EVENT*** IF DIFFERENT SUB-REGIONS; AGAIN, 'MAINLY' RULE FOR TRUE BORDERLINE CASES)

6, 7a **(And) what kind of fishing/etc did you do that day? Any others?** (INITIAL PROBE FOR TARGET/ METHOD SPLITS, THEN ASK AS APPROP:) **Were you fishing for anything in particular or not? Anything else?** (PROBE FOR AND RECORD MAIN/2ND MAIN TARGET SPECIES AS APPROP AND APPLY SPECIES CODE/S FROM Q10; CODES 'W' AND 'X' MAINLY FOR SUB-REGIONS 1 AND 2; ***SPLIT EVENT*** IF INITIAL TARGETING CODE/S CHANGE)

'

Continued/…………

Q'n No:

7a **(And) did you use bait, lures … (or both)?** (PROBE ALL OTHER METHODS AS REQUIRED; ***SPLIT EVENT*** IF MORE THAN ONE CODE, BUT NOT IF JUST CODES 1 & 2 [CODE 3 APPLIES]; NOTE: PASSIVE POT/TRAP (CODE 5) IS THE MOST COMMON POT/TRAP FOR CRABS/ETC – AND DIFFERS FROM CODE 6, WHERE MOVEMENT IS REQUIRED ('ACTIVE')

7b (IF POTS OR NETS USED [CODES 5, 6, 9, OR 10] IN Q7a], ASK:) **(And) how many pots/etc did you use (that day)?** (***SPLIT EVENT*** IF NUMBER OF POTS OR NETS CHANGED)

7c (IF EFFORT SHARING POSSIBLE PER Q7a/b, ASK:) **(And) how many people were actually fishing/ etc with you?** (PROBE AND RECORD NUMBER ACTUALLY 'INVOLVED' [INCL. ANY NON-DIARISTS AND MEMBERS OF OTHER H'HOLDS]; AMEND Q'S 1 AND 7b AS APPROP; AND ALERT Q10 FOR SHARED CATCH)

8a **(And) did you fish/etc from a boat, the shore (… or both)?** **(NO *SPLIT EVENT*)**

8b (IF BOAT PER Q8a, ASK:) **(And) whose boat was it?** (PROBE FURTHER AS APPROP: CHARTER = SKIPPERED, HIRE = SELF-DRIVE; ***SPLIT EVENT*** IF BOAT TYPE DIFFERENT [RARE])

8c (IF SHORE PER Q8a, ASK:) **(And) did you fish from a man-made structure of any kind, a beach, the rocks …or some other shore type (e.g. river bank)?** (IF MORE THAN ONE CODE, PROBE/RECORD MAIN CODE; IF ROCKS, ALERT/PROBE BREAKWALL [CODE 3]; CODES 1 AND 2 = OCEAN WATERS ONLY; **NO *SPLIT EVENT*)**

9 **(And) when did you actually start fishing/etc ..... (that day/for/etc)?** (LINES/ETC IN THE WATER) **(And) when did you finish?** (LINES/ETC OUT OR LAST CHECK, IF CONTINUOUS PASSIVE GEAR USED; IF ANY EVENT FOR THIS CONTACT IS INCOMPLETE, FLAG 'OPEN EVENT' IN Q11 COMMENTS AND ALSO ON DSC) **(And) during this time, did you have any breaks from fishing/etc?** (EITHER RESPONDENT OR GEAR; PROBE/ADD/RECORD; IF NIL BREAKS, ENTER 'DASH')

10 **(And) did you (personally) catch anything (that day) ...?** (PROBE NUMBER CAUGHT AND KEPT BY SPECIES; CARE WITH SHARED CATCHES/JOINT EFFORT). **Are you certain that's what it was/they were?** (PROBE ID GUIDE CHECKED, THEN ASK:) **Did you (personally) catch anything else (that day) that you released?** (PROBE NUMBER CAUGHT AND RELEASED BY SPECIES) (**And) why did you release** (EG) **the two flathead?** (I.E. NO. X EACH SPECIES; AVOID PROBING OTHER THAN E.G. ..) **Were all of them too small?** (ABSOLUTELY NO PROMPTING; THEN CLASSIFY NO. RELEASED BY REASON FOR EACH SPECIES [SEE BELOW AND FULL DEFINITIONS IN MANUAL]; IF NIL KEPT/ RELEASED, CIRCLE CODE 'x')

TS = too small (personal preference), as opposed to <S = under-size (legal limit)

TM = too many (personal preference), as opposed to >N = over legal bag/possession limit

CR = catch and release (personal preference) – only applies to the total daily catch of a species (i.e. none kept)

OT without further specification = either unwanted/poor eating quality species or berried female crabs/lobsters (i.e. with eggs). For other OT cases (with specification) see interviewer manual (e.g. too big, too few etc)

11 COMMENTS: PLEASE ONLY RECORD HERE INFORMATION OF RELEVANCE TO OFFICE CODING – I.E. NOT OPININONS/ETC THAT CAN BE DEFERRED TO WASH-UP SURVEY (AT END OF DIARY PERIOD). IF EXTRA SPACE NEEDED, CIRCLE CODE 'x' AND RECORD ON BACK OF PAGE

C AFTER LAST EVENT RECORDED

- **(And) do you (or any other family/household member) have any fishing trips planned for the rest of this month/etc?** (MAKE GENERAL/SPECIFIC APPOINTMENT AS APPROP; IF NO PLANS, MONTHLY RULE INITIALLY/GENERALLY; ONLY IF NECESSARY, BI-MONTHLY CALLS ACCEPTABLE FOR ADAMANT/ INFREQUENT FISHERS; RECORD LAST EVENT OR ANY 'OPEN' EVENTS ON DSC FOR NEXT CONTACT)

© 2013 Fisheries NSW (DPI) and Kewagama Research

SURVEY OF RECREATIONAL FISHING – 2013/14 **EVENT SHEET©** **Sample No:** N/L **Event No:**

1. Person Numbers:

☐ ☐ ☐ ☐ ☐

2. Start date

(Day/Mth) ☐

End (if diff, plus ...) ☐

3. Data source

Personal/diarised	1
Personal/not diarised	2
Proxy/diarised	3
Proxy/not diarised	4

4. Fishing Region *(split)*

……………………………………

…………………………… ☐

…………………………....

5. Sub-region *(split)*

Offshore (>5km)	1
Inshore (<5km)	2
River/estuary (SW)	3
River/stream (FW)	4
Lake/dam (FW) - public	5
Lake/dam (FW) - private	6

6(a/b) Targeting *(split)*

Species/group	Main	2nd
……………….		
……………….		
No specific target		
- surface	w	w
- bottom	x	x
- all other	y	y
No 2nd target		z

7(a) Method *(split/*go Q8)*

Lines - bait*	1
Lines - lure/jig/fly*	2
Lines - both*	3
*Lines - set /passive**	*4*
Pot/trap - passive *(go b,c)*	5
Pot/trap - active *(go b,c)*	*6*
*Net - cast**	*7*
Net – drag/seine *(go b,c)**	8
Net – gill/set (go b,c)	*9*
Net – scoop/push *(go b,c)*	10
Spearfishing - diving*	11
Other spearing - surface*	12
Other diving	
- scuba/surface. air*	13
- snorkel*	14
- both*	15
Hook/pump/rake/spade*	16
Other hand collecting*	17
Other (specify) …………………	☐

(b) No. of Pots/Nets ☐

(c) No. of Persons ☐
(shared effort only)

8(a) Platform

Boat *(after Q8b, go to Q9)*	1
Shore *(go to Q8c)*	2
Both	3

(b) Boat type *(split)*

Private	1
Hire	2
Charter	3

(c) Shore type (main)

Ocean beach (Q5 < 3)	1
Ocean rocks (Q5 < 3)	2
Man-made structure	3
Other/natural shore (Q5 > 2)	4

9. Times

Start	(24 hr. clock)	:
Finish	(24 hr. clock)	:
Breaks	(hrs. & mins.)	:

10. Catch

SPECIES		No. Caught & Kept/Released	No. Released x Reason TS <S TM >N CR OT
Batfish/butter br'm	371	……./…….	…../…../…../…../…../…..
Bream-all	14	……./…….	…../…../…../…../…../…..
Catfish-eeltail (both)	19	……./…….	…../…../…../…../…../…..
Catfish-forktail (both)	18	……./…….	…../…../…../…../…../…..
Cod-red rock	30	……./…….	…../…../…../…../…../…..
Cod-other (SW)	31	……./…….	…../…../…../…../…../…..
Dart-all	35	……./…….	…../…../…../…../…../…..
Drummer-black	134	……./…….	…../…../…../…../…../…..
Drummer-silver	39	……./…….	…../…../…../…../…../…..
Eel-all (both)	43	……./…….	…../…../…../…../…../…..
Flathead-blue./sand	57	……./…….	…../…../…../…../…../…..
Flathead-dusky	56	……./…….	…../…../…../…../…../…..
Flathead-tiger	58	……./…….	…../…../…../…../…../…..
Flounder/sole-all	61	……./…….	…../…../…../…../…../…..
Garfish-all	65	……./…….	…../…../…../…../…../…..
Groper-blue	69	……./…….	…../…../…../…../…../…..
Gurnard-all	73	……./…….	…../…../…../…../…../…..
Kingfish-yellowtail	84	……./…….	…../…../…../…../…../…..
Leatherjacket-all	87	……./…….	…../…../…../…../…../…..
Luderick/blackfish	91	……./…….	…../…../…../…../…../…..
Mackerel-blue/slimy	92	……./…….	…../…../…../…../…../…..
Morwong-blue/grey	108	……./…….	…../…../…../…../…../…..
Morwong-red	111	……./…….	…../…../…../…../…../…..
Mullet-all	117	……./…….	…../…../…../…../…../…..
Mulloway/jewfish	118	……./…….	…../…../…../…../…../…..
Perch-pearl	123	……./…….	…../…../…../…../…../…..
Pigfish	124	……./…….	…../…../…../…../…../…..
Rays-shovelnose	129	……./…….	…../…../…../…../…../…..
Rays-other	130	……./…….	…../…../…../…../…../…..
Redfish/nannygai	132	……./…….	…../…../…../…../…../…..
Salmon-Aust.	136	……./…….	…../…../…../…../…../…..
Sergeant baker	143	……./…….	…../…../…../…../…../…..
Shark-whaler/bull	155	……./…….	…../…../…../…../…../…..
Snapper-pink	162	……./…….	…../…../…../…../…../…..
Sweep-all	175	……./…….	…../…../…../…../…../…..

(continued ….)		Kept/Released	TS <S TM >N CR OT
Tailor	177	……./…….	…../…../…../…../…../…..
Tarwhine	178	……./…….	…../…../…../…../…../…..
Teraglin	179	……./…….	…../…../…../…../…../…..
Toad/pufferfish	180	……./…….	…../…../…../…../…../…..
Trevally-silver	185	……./…….	…../…../…../…../…../…..
Trumpeter/grunter	374	……./…….	…../…../…../…../…../…..
Whiting-sand	212	……./…….	…../…../…../…../…../…..
Whiting-school	355	……./…….	…../…../…../…../…../…..
Whiting-trumpeter	213	……./…….	…../…../…../…../…../…..
Wrasse-maori	219	……./…….	…../…../…../…../…../…..
Wrasse-other	220	……./…….	…../…../…../…../…../…..
Yellowtail/scad	222	……./…….	…../…../…../…../…../…..
SMALL BAITFISH	299	……./…….	…../…../…../…../…../…..
Bass-Aust. (FW)	224	……./…….	…../…../…../…../…../…..
Carp-all (FW)	226	……./…….	…../…../…../…../…../…..
Cod-murray (FW)	228	……./…….	…../…../…../…../…../…..
Perch-golden (FW)	231	……./…….	…../…../…../…../…../…..
Perch-redfin (FW)	235	……./…….	…../…../…../…../…../…..
Perch-silver (FW)	233	……./…….	…../…../…../…../…../…..
Trout-brown (FW)	242	……./…….	…../…../…../…../…../…..
Trout-rainbow (FW)	243	……./…….	…../…../…../…../…../…..
Crab-blue swimmer	249	……./…….	…../…../…../…../…../…..
Crab-mud	250	……./…….	…../…../…../…../…../…..
Pipis	273	……./…….	…../…../…../…../…../…..
Prawns-all (SW)	260	……./…….	…../…../…../…../…../…..
Prawns/shrimp (FW)	346	……./…….	…../…../…../…../…../…..
Sthn. Calamari	286	……./…….	…../…../…../…../…../…..
Squid-other	285	……./…….	…../…../…../…../…../…..
Yabbies (FW)	263	……./…….	…../…../…../…../…../…..
Yabbies/nip'rs (SW)	266	……./…….	…../…../…../…../…../…..
Worms-beach	245	……./…….	…../…../…../…../…../…..
……………………(	)	……./…….	…../…../…../…../…../…..
……………………(	)	……./…….	…../…../…../…../…../…..

NIL CATCH/RELEASE **x**

11. Comments: ………………………….…………… **x** (overleaf)

© 2013 Fisheries NSW (DPI) and Kewagama Research
